# Ten-year analysis of microbiological profile and antibiotic sensitivity for bacterial keratitis in Korea

**DOI:** 10.1371/journal.pone.0213103

**Published:** 2019-03-01

**Authors:** Yongseok Mun, Mee Kum Kim, Joo Youn Oh

**Affiliations:** 1 Department of Ophthalmology, Seoul National University Hospital, Seoul, Republic of Korea; 2 Laboratory of Ocular Regenerative Medicine and Immunology, Seoul Artificial Eye Center, Seoul National University Hospital Biomedical Research Institute, Seoul, Republic of Korea; Rutgers New Jersey Medical School, UNITED STATES

## Abstract

**Purpose:**

To investigate the risk factors, microbiological profiles, antibiotic susceptibility patterns, and treatment outcome in patients with bacterial keratitis at a Korean tertiary hospital.

**Methods:**

A retrospective chart review was performed of patients who were diagnosed with infectious keratitis and underwent corneal scrapings for cultures at Seoul National University Hospital between 2007 and 2016. Demographics, clinical characteristics, microbiological data, antibiotic resistance and sensitivity, and treatment outcome were collected.

**Results:**

Out of 129 scrapings, bacteria were isolated in 101 samples (78.3%). The most frequent isolates were coagulase-negative *Staphylococci* (CNS) (15.9%), *Staphylococcus aureus* (12.1%) and *Pseudomonas aeruginosa* (10.3%). All gram-positive isolates were sensitive to vancomycin, but methicillin resistance was found in 29.4% of CNS and 15.4% of *Staphylococcus aureus*. All gram-negative isolates were susceptible to ceftazidime and carbapenem while 11.5%, 3.3% and 2.8% of gram-negative isolates were resistant to gentamicin, tobramycin and amikacin, respectively. Ciprofloxacin resistance was observed in 10.3% of gram-positive isolates and 8.8% of gram-negative isolates. No significant changes were observed in profiles of microbial isolates and antibiotic sensitivity over time. Eight eyes of 101 eyes (7.9%) eventually underwent evisceration for infection control. The use of topical glaucoma medication (*p* = 0.006) and history of ocular surgery (*p* = 0.019) were significant risk factors related to evisceration.

**Conclusions:**

CNS, S*taphylococcus aureus* and *Pseudomonas aeruginosa* were the most common microorganisms responsible for bacterial keratitis. The duo-therapy using vancomycin and ceftazidime should be considered for empirical treatment until the culture and sensitivity results become available.

## Introduction

Infectious keratitis is a significant cause of corneal opacification and vision loss [1–4]. Prompt treatment with appropriate antimicrobial eye drops is critical to avoid the sequelae in patients with infectious keratitis. For selection of the effective antimicrobial agents, identification of causative microorganisms and screening for antibiotic sensitivity are necessary from corneal scrapings in individual patients [5]. However, corneal culture service is not always available in clinical setting, and bacterial growth on culture plates takes several days and weeks. Therefore, empirical therapy with broad-spectrum antibiotics should be initiated before culture results and remained the primary treatment modality for infectious keratitis [6–8].

Recently, antimicrobial resistance has emerged as a major issue in infection. In ophthalmology, the incidence of infectious keratitis has risen in the last decade, partially due to an increasing number of contact lens users and immune-compromised patients [9–13]. Also, the changes have been reported in microbial compositions responsible for infectious keratitis and antibiotic resistance patterns [13–24]. Since the causes of infectious keratitis largely vary depending on climate and geography [13–24], it is important to analyze regional microbial profiles and antibiotic susceptibility patterns for evidence-based selection of empirical treatment regimen.

In this study, we investigated the profiles of bacteria isolated from corneal cultures in patients with infectious keratitis, the antibiotic susceptibility and resistance patterns, and treatment outcome over a 10-year period at Seoul National University Hospital, a tertiary referral hospital in South Korea.

## Patients and methods

This study was approved by the Institutional Review Board at Seoul National University Hospital. A retrospective analysis of medical charts was conducted in patients who were diagnosed with infectious keratitis and underwent corneal scraping and culture from January 1, 2007 and December 31, 2016 at Seoul National University Hospital in South Korea. Noncorneal samples or swab culture in transport medium were excluded from analysis. Nonbacterial infectious keratitis such as fungal, viral or acanthamoebal infection were also excluded.

The following data were collected from each medical record: age, gender, laterality of the affected eye, ethnicity, immune-compromising disorders such as diabetes mellitus, contact lens use, glaucoma, serious ocular surface disease (Stevens-Johnson syndrome, etc.), history of ocular surgery or trauma, use of topical corticosteroids or anti-glaucoma medications, microorganisms isolated from corneal culture, antibiotic sensitivity and resistance, treatment, visual acuity, and significant complications.

Corneal scrapes were obtained as per the standardized protocol. A sterile, disposable blade (#15) was used for scraping, and the scrapes were immediately inoculated onto the following culture plates: blood agar, chocolate agar, Sabouraud dextrose agar, Sabouraud dextrose agar with chloramphenicol, and brain heart infusion broth. The plates were then incubated at 37°C in 5% CO_2_. Culture positivity was defined by an experienced laboratory staff as the growth of microorganisms along the line of inoculation on at least one solid medium. The techniques for inoculation and isolation were consistent throughout the study period.

Antimicrobial identification and susceptibility test were performed by an automated Vitek 2 system (bioMérieux, Inc., Marcy l’Etoile, France). Isolates were categorized as sensitive, intermediate, or resistant to the tested antibiotics. The tested antibiotics included vancomycin, cefazolin, cefuroxime, chloramphenicol, oxacillin, penicillin, ciprofloxacin, levofloxacin, moxifloxacin, carbapenem (meropenem and imipenem), tobramycin, ceftazidime, amikacin, and gentamicin.

For analysis, the isolated microorganisms were divided into four categories: gram-positive cocci, gram-positive bacilli, gram-negative cocci, and gram-negative bacilli. Also, the study period was divided into the two groups (2007–2011 and 2012–2016) to evaluate the changes of microbial and antibiotic resistance profiles over time.

Statistical analysis was performed using SPSS software, version 11 (SPSS Inc., Chicago, IL). The Chi-square and Fisher’s exact tests were used to compare independent variables and assess statistical significance. *P* value < 0.05 was considered significant.

## Results

### Patient demographics and clinical characteristics

A total of 129 corneal scrape specimens (129 eyes, 129 patients) were analyzed. All patients had unilateral eye involvement. Of these, bacteria were isolated in 101 eyes of 101 patients (78.3%). Fungi were isolated in 10 eyes (7.8%), and 18 eyes had negative cultures.

Summarized in Table 1 were demographical and clinical characteristics of 101 patients who had positive cultures for bacteria. All patients were Korean. The mean age of patients with positive bacterial cultures was 57.4 ± 19.9 years (range 0–83). Fifty two (51.5%) were male and 49 (48.5%) female. The right eye was affected in 36 patients (35.6%) and the left eye in 65 (64.4%).

**Table 1:** Demographics and clinical characteristics of patients with bacterial keratitis.

	Total	2007–2011	2012–2016	*P* value
Number of eyes (%)	101	39 (38.6%)	62 (61.4%)	
Age (years)	57.4 ± 19.9	53.4 ± 22.2	59.9 ± 18.2	0.110
Sex (male:female)	52:49	23:16	29:33	0.232
Diabetes mellitus	17 (16.8%)	10 (25.6%)	7 (11.3%)	0.061
Immune-compromised status (SJS, etc.)	12 (11.9%)	4 (10.3%)	8 (12.9%)	0.762
Contact lens wear	12 (11.9%)	4 (10.3%)	8 (12.9%)	0.762
Topical anti-glaucoma medication	29 (28.7%)	11 (28.2%)	18 (29.0%)	0.929
Topical corticosteroids	39 (38.6%)	22 (56.4%)	17 (27.4%)	0.004
Ocular surgery history	60 (59.4%)	24 (61.5%)	36 (58.1%)	0.729
PK Cataract surgery Glaucoma surgery Pterygium excision Others	30 (29.7%) 34 (33.7%) 10 (9.9%) 1 (1%) 8 (7.9%)	16 (41.0%) 14 (35.9%) 2 (5.1%) 0 2 (5.1%)	14 (22.6%) 20 (32.3%) 8 (12.9%) 1 (1.6%) 6 (9.7%)	
Ocular trauma history	8 (7.9%)	2 (5.1%)	6 (9.7%)	0.480

PK: penetrating keratoplasty, SJS: Stevens-Johnson syndrome

The potential risk factors associated with infectious keratitis are demonstrated in Table 1. Diabetes mellitus was present in 17 patients (16.8%), and other immune- compromised diseases such as Stevens-Johnson syndrome were found in 12 (11.9%). Twelve patients (11.9%) were contact lens wearers. Remarkably, a high percentage of patients used topical corticosteroids (39 patients, 38.6%) or anti-glaucoma medications (29 patients, 28.7%) at the time of infection. Also, 60 eyes (59.4%) had a history of ocular surgery, of which 30 eyes (29.7%) had penetrating keratoplasty (PK). Only 8 (7.9%) patients reported a previous ocular trauma in an affected eye.

When each variable was compared between the two study periods (2007–2011 and 2012–2016), the number of eyes that received topical corticosteroids was significantly lower in the 2012–2016 study period than in the 2007–2011 group (*p* = 0.004, Table 1). Otherwise, there was no statistical difference in other clinical factors between the two groups (Table 1).

### Causative bacteria

A total of 107 bacteria were identified in 101 culture-positive specimens (two bacteria simultaneously isolated in one specimen in 6 eyes). Sixty nine (64.5%) were gram-positive and 38 (35.5%) were gram-negative. The detailed information of isolated bacteria is listed in Table 2. The most common bacteria were coagulase-negative *Staphylococci* (CNS) (17 eyes, 15.9%) followed by *Staphylococcus aureus* (13 eyes, 12.1%), *Pseudomonas aeruginosa* (11 eyes, 10.3%), *Streptococcus pneumoniae* (9 eyes, 8.4%), *Streptococcus viridans* (6 eyes, 5.6%), and *Corynebacterium species* (6 eyes, 5.6%). There was no significant difference in the proportions of gram-positive and gram-negative microorganisms between the 2007–2011 and 2012–2016 study periods (Table 2).

**Table 2:** Identification of bacteria isolated from cultures of corneal scraping.

	Total	2007–2011	2012–2016
**Total**	107	42	65
**G (+) cocci**	60 (56.1%)	26 (61.90%)	34 (52.3%)
Coagulase-negative staphylococci (CNS)	17 (15.9%)	10(23.8%)	7 (10.8%)
*Staphylococcus epidermidis*	9	6	3
*Staphylococcus auricularis*	1	0	1
*Staphylococcus cohnii* ss. *urealyticus*	1	1	0
*Staphylococcus hominis* ss.	1	1	0
*Staphylococcus saprophyticus*	1	0	1
*Staphylococcus simulans*	1	1	0
*Staphylococcus warneri*	2	1	1
CNS, unspecified	1	0	1
*Staphylococcus aureus*	13 (12.1%)	7 (16.7%)	6 (9.2%)
*Streptococcus pneumoniae*	9 (8.4%)	4 (9.5%)	5 (7.7%)
Viridans group streptococci	6 (5.6%)	3 (7.1%)	3 (4.6%)
*Streptococcus sanguinis*	3 (2.8%)	0 (0%)	3 (4.6%)
*Streptococcus parasanguinis*	2 (1.9%)	0 (0%)	2 (3.1%)
*Streptococcus constellatus* ssp.	1 (0.9%)	0 (0%)	1 (1.5%)
*Streptococcus dysgalactiae* ss.	1 (0.9%)	0 (0%)	1 (1.5%)
*Streptococcus pyogenes*	1 (0.9%)	1 (2.4%)	0 (0%)
*Enterococcus faecalis*	1 (0.9%)	0 (0%)	1 (1.5%)
*Micrococcus luteus*	1 (0.9%)	0 (0%)	1 (1.5%)
*Micrococcus* species	4 (3.7%)	1 (2.4%)	3 (4.6%)
*Dermacoccus nishinomiyaensis*	1 (0.9%)	0 (0%)	1 (1.5%)
**G (+) bacilli**	9 (8.4%)	2 (4.8%)	7 (10.8%)
*Corynebacterium* species	6 (5.6%)	2 (4.8%)	4 (6.2%)
*Brevibacillus* species	2 (1.9%)	0 (0%)	2 (3.1%)
*Bacillus* species	1 (0.9%)	0 (0%)	1 (1.5%)
**G (-) cocci**	3 (2.8%)	2 (4.8%)	1 (1.5%)
*Moraxella* species	2 (1.9%)	1 (2.4%)	1 (1.5%)
*Moraxella catarrhalis*	1 (0.9%)	1 (2.4%)	0 (0%)
**G (-) bacilli**	35 (32.7%)	12 (28.6%)	23 (35.4%)
*Pseudomonas aeruginosa*	11 (10.3%)	4 (9.5%)	7(10.8%)
*Acinetobacter baumannii*	5 (4.7%)	1 (2.4%)	4 (6.2%)
*Serratia marcescens* ss.	4 (3.7%)	3 (7.1%)	1 (1.5%)
*Sphingomonas paucimobilis*	2 (1.9%)	0 (0%)	2 (3.1%)
*Achromobacter xylosoxidans*	1 (0.9%)	0 (0%)	1 (1.5%)
*Achromobacter denitrificans*	1 (0.9%)	0 (0%)	1 (1.5%)
*Acinetobacter lwoffii*	1 (0.9%)	1 (2.4%)	0 (0%)
*Aeromonas hydrophila*	1 (0.9%)	0 (0%)	1 (1.5%)
*Haemophilus influenzae*	1 (0.9%)	1 (2.4%)	0 (0%)
*Moraxella* species	1 (0.9%)	0 (0%)	1 (1.5%)
*Morganella morganii* ss.	1 (0.9%)	0 (0%)	1 (1.5%)
*Paenibacillus* spp.	1 (0.9%)	0 (0%)	1 (1.5%)
*Pantoea agglomerans*	1 (0.9%)	1 (2.4%)	0 (0%)
*Pantoea* species	1 (0.9%)	0 (0%)	1 (1.5%)
*Providencia rettgeri*	1 (0.9%)	0 (0%)	1 (1.5%)
*Pseudomonas mendocina*	1 (0.9%)	0 (0%)	1 (1.5%)
*Pseudomonas putida*	1 (0.9%)	1 (2.4%)	0 (0%)

### Antibiotic susceptibility

The antibiotic susceptibility for isolated organisms is demonstrated in Table 3. All gram-positive bacteria were sensitive to vancomycin, whereas 53.8% of gram-positive isolates were sensitive to cefazolin and 53.3% were sensitive to oxacillin (Table 3). There was no significant difference in the rate of oxacillin resistance among gram-positive isolates between the 2007–2011 and 2012–2016 periods (*p* = 0.881). The methicillin-resistant *Staphylococcus aureus* (MRSA) comprised 15.4% of *Staphylococcus aureus* isolated, and the percentage of methicillin-resistant CNS (MRCNS) was 29.4% of CNS. There was no difference in the percentage of MRSA (*p* = 0.379) or MRCNS (*p* = 0.076) between the two study periods. The rate of resistance to ciprofloxacin was 10.3% among gram-positive isolates with no difference between the first and second study periods (*p*>0.999). Most of gram-positive bacteria were sensitive to levofloxacin and moxifloxacin (95.8% and 93.8%, respectively) (Table 3).

**Table 3:** Antibiotic susceptibility of isolated bacteria. Number of eyes (%).

	Gram-positive			Gram-negative		
	Total	2007–2011	2012–2016	Total	2007–2011	2012–2016
Vancomycin	67/67 (100%)	28/28 (100%)	39/39 (100%)	1/1 (100%)	N/A	1/1 (100%)
Cefazolin	7/13 (53.8%)	7/13 (53.8%)	N/A	1/4 (25.0%)	1/4 (25.0%)	N/A
Cefuroxime	8/18 (44.4%)	7/14 (50.0%)	1/4 (25.0%)	3/6 (50.0%)	2/5 (40.0%)	1/1 (100%)
Chloramphenicol	37/43 (86.0%)	17/20 (85%)	20/23 (87.0%)	2/2 (100%)	1/1 (100%)	1/1 (100%)
Oxacillin	16/30 (53.3%)	10/18 (55.5%)	6/12 (50.0%)	N/A	N/A	N/A
Penicillin	9/41 (22.0%)	2/21 (9.5%)	7/20 (35%)	N/A	N/A	N/A
Ciprofloxacin	26/29 (89.7%)	14/16 (87.5%)	12/13 (92.3%)	31/34 (91.2%)	13/14 (92.9%)	18/20 (90.0%)
Levofloxacin	23/24 (95.8%)	5/5 (100%)	18/19 (94.7%)	8/8 (100%)	8/8 (100%)	N/A
Moxifloxacin	15/16 (93.8%)	4/4 (100%)	11/12 (91.7%)	N/A	N/A	N/A
Meropenem	9/16 (56.3%)	8/14 (57.1%)	1/2 (50.0%)	31/31 (100%)	11/11 (100%)	20/20 (100%)
Imipenem	14/28 (50.0%)	8/16 (50.0%)	6/12 (50.0%)	36/36 (100%)	14/14 (100%)	22/22 (100%)
Tobramycin	1/1 (100%)	1/1 (100%)	N/A	29/30 (96.7%)	13/13 (100%)	16/17 (94.1%)
Ceftazidime	N/A	N/A	N/A	34/34 (100%)	13/13 (100%)	21/21 (100%)
Amikacin	0/1 (0)	N/A	0/1 (0)	35/36 (97.2%)	13/13 (100%)	22/23 (95.7%)
Gentamicin	19/28 (67.9%)	10/16 (62.5%)	9/12 (75.0%)	23/26 (88.5%)	10/10 (100%)	13/16 (81.3%)

N/A: not available

All gram-negative bacteria were sensitive to ceftazidime and carbapenem (meropenem and imipenem), and most of gram-negative isolates were susceptible to tobramycin and amikacin (96.7% and 97.2%, respectively) (Table 3). However, 11.5% of gram-negative isolates were resistant to gentamicin. Ciprofloxacin resistance was observed in 8.8% of gram-negative isolates. There was no significant difference in the resistance rates of gram-negative isolates to any tested antibiotics between the first and second study periods. Notably, *Pseudomonas* species, the most frequently isolated gram-negative bacteria, were sensitive in every case to ceftazidime, amikacin, ciprofloxacin, and carbapenem.

### Treatment outcome

The visual acuity improved in 47.4% of eyes after treatment and remained stationary in 32.6%. However, the vision worsened in 20.0% despite treatment. The visual outcome was not different between patients with gram-positive bacterial keratitis and those with gram-negative bacterial keratitis (Table 4).

**Table 4:** Visual outcome after treatment. Number of eyes (%).

Visual acuity post- vs. pre-treatment	Gram-positive	Gram-negative	Total	*P* value
Improved	31 (50.8%)	14 (41.2%)	45 (47.4%)	0.623
No change	18 (29.5%)	13 (38.2%)	31 (32.6%)	
Worsened	12 (19.7%)	7 (20.6%)	19 (20.0%)	
Total	61	34	95	

Because of insufficient response to antibiotics treatment, 6 eyes (5.9%) received PK and 8 eyes (7.9%) underwent evisceration. Additional analysis revealed that the use of topical anti-glaucoma medications and history of ocular surgery were the factors significantly associated with evisceration (*p* = 0.006 and 0.019, respectively) (Table 5). Of note, all 8 eyes that required evisceration had a previous history of ocular surgery in an infected eye as follows: PK + limbal allografts (n = 1), PK + cataract surgery + trabeculectomy (n = 3), PK + pars plana vitrectomy (n = 1), pars plana vitrectomy + cataract surgery (n = 1), glaucoma surgery (n = 1), corneal foreign body removal + cataract surgery (n = 1). However, other factors such as age, time of presentation to our center after the symptom onset, presence of immunocompromised conditions, use of topical corticosteroids, or history of ocular trauma were not significantly different between patients who had evisceration and those who did not (Table 5). Bacterial isolates from 8 eyes who required evisceration were *Pseudomonas aeruginosa* (n = 3), *Acinetobacter baumannii* (n = 1), *Providencia rettgeri* (n = 1), *Streptococcus pneumoniae* (n = 1), *Staphylococcus warneri* (n = 1), and the viridans group streptococci (n = 1).

**Table 5:** Risk factors associated with evisceration. Number of eyes (%).

	Evisceration	No evisceration	*P* value
Number of eyes	8	93	
Age (years)	56.6 ± 20.4	66.1 ± 12.1	0.204
Immunocompromised disease (diabetes mellitus, SJS, etc.)	3 (37.5%)	26 (27.9%)	0.686
Topical glaucoma medication	6 (75.0%)	23 (24.7%)	0.006
Topical corticosteroids	5 (62.5%)	34 (36.6%)	0.255
History of ocular surgery	8 (100%)	52 (55.9%)	0.019
History of ocular trauma	2 (25.0%)	6 (6.5%)	0.121
Gram (+) / Gram (-)	3 / 5	63 / 30	0.121

SJS: Stevens-Johnson syndrome

## Discussion

Proper diagnosis and treatment of bacterial keratitis are essential to achieve resolution of infection and minimize damage to the cornea. The mainstay in diagnosis is culture of corneal samples, and an additional assay to test antibiotics sensitivity *in vitro* is valuable to select effective antibiotics against the causative microorganism(s) in individual patients [5]. Since there is high variability in microbial isolates and their antibiotic resistance according to geographic location, population, and with time [13–24], it is important to obtain the regional data on microbial trends. We herein analyzed the microbial spectrum and antibiotic susceptibility patterns in patients with infectious keratitis over the last 10 years at a tertiary referral hospital in South Korea.

The overall yield of bacteria-positive cultures in our center was 78.3%, which is similar to recent studies from Vancouver and Sydney (75%) [13, 14] and higher compared with other studies (23.7% to 61.5%) [15–24]. The most commonly isolated organism was CNS, which accounted for 15.9% of total bacterial isolates and 24.6% of gram-positive isolates. The second common organism was *Staphylococcus aureus* (12.1% of total isolates) followed by *Pseudomonas aeruginosa* (10.3% of total isolates). *Pseudomonas aeruginosa* was the most common gram-negative isolate and accounted for 28.9% of gram-negative isolates. In accordance with our findings, CNS was identified as the most common isolate in bacterial keratitis in the majority of recent series [13, 14, 16, 18, 19, 21, 22], whereas some studies found *Pseudomonas aeruginosa* to be the most prevalent pathogen, presumably associated with contact lens use [15, 20, 24]. Although several studies found a significant increase in the percentage of gram-negative isolates over time [14–16, 18, 22], our study did not observe such trend because the prevalence of gram-negative isolates remained stable during the study period (33.3% in 2007–2011 and 36.9% in 2012–2016). Interestingly, the isolation rate of bacilli in corneal specimens increased from 33.3% (2007–2011) to 46.2% (2012–2016) in our study, while the rate of cocci isolation declined from 66.7% (2007–2011) to 53.8% (2012–2016). However, this difference did not reach statistical significance (*p* = 0.188).

Increasing resistance to antibiotics in ocular infection has been observed in other series. Methicillin resistance, a marker for multi-drug resistance and virulent course of infection, was reported in 1.3% to 45% of *Staphylococcus aureus* and in 3% to 53.7% of CNS in patients with infectious keratitis [13, 14, 16–18, 24]. In our patients, MRSA was present in 15.4% of *Staphylococcus aureus*, and MRCNS was found in 29.4% of CNS. There was no significant increasing trend over time. All gram-positive bacteria including methicillin-resistant strains were sensitive to vancomycin. Although a recent study from Mexico noted an increased resistance of *Pseudomonas aeruginosa* to ceftazidime up to 74% [18], we found that all gram-negative isolates including *Pseudomonas aeruginosa* were susceptible to ceftazidime. Moreover, *Pseudomonas* species, the most frequently isolated gram-negative bacteria, were all sensitive to amikacin and ciprofloxacin in our patients. This finding is in contrast to the concern that *Pseudomonas* species are developing antibiotic resistance [25, 26]. The resistance to ciprofloxacin, one of fluoroquinolone often used in monotherapy, was found in 10.3% and 8.8% of gram-positive and negative isolates, respectively, and these figures are comparable to other reports [14, 15, 18, 20, 24]. Based on these findings, we are currently using a combination of vancomycin and ceftazidime as the initial empirical therapy for patients with suspected bacterial keratitis and for the cases that were pre-treated at the community with fluoroquinolones but have not responded to the treatment.

Despite antibiotics treatment, evisceration was eventually performed in 7.9% of patients due to uncontrolled infection, and the rate of evisceration is higher than reported previously (1.9% to 4.1%) [27, 28]. This may be partly due to the fact that a high percentage of our patients had pre-existing ocular and systemic morbidities. In fact, 28.7% of our patients were using anti-glaucoma medications at the time of infection, and 38.6% were using topical corticosteroids. Also, 59.4% of patients had previous surgery in the affected eye, where 29.7% had PK and 9.9% had glaucoma surgery. The previous history of ocular surgery and the use of topical anti-glaucoma medications were found to be significant factors associated with evisceration in our patients.

Our study has several limitations. First, as mentioned above, the study was conducted in a tertiary care, university referral center; therefore, it may not be possible to generalize our findings to other regions or populations. Second, we cannot exclude the possibility that the positive cultures in some of the cases were caused by contaminant sources or non-pathogenic commensals from the ocular surface such as CNS. It is also possible that the failure of bacterial growth on culture plates in some cases might be due to insufficient corneal samples although our culture positivity rate was high (78.3%). Third, *in vitro* testing of antibiotic susceptibility and resistance does not always correlate with clinical response of infectious microorganisms to given antibiotics. Moreover, we did not test gram-negative isolates against moxifloxacin, the fourth generation fluoroquinolone, which is commonly used as the community-based empirical treatment of corneal ulcers. Still, approximately 90% of both gram-positive and negative isolates were susceptible to ciprofloxacin and levofloxacin that have higher mean inhibitory concentrations than moxifloxacin.

In conclusion, our data demonstrate that CNS, S*taphylococcus aureus* and *Pseudomonas aeruginosa* were the most common microorganisms responsible for bacterial keratitis in South Korea. The spectrums of bacteria and antibiotic sensitivity profiles have not changed significantly between 2007 and 2016. All isolated bacteria were covered by the combined therapy with vancomycin and ceftazidime.

## Supporting information

S1 File Raw data on patient demographics, risk factors, isolated microorganisms, antibiotic sensitivity, and visual outcome. (XLSX)
